# Redox-Activation of Neutrophils Induced by Pericardium Scaffolds

**DOI:** 10.3390/ijms232415468

**Published:** 2022-12-07

**Authors:** Irina I. Vlasova, Shakir K. Suleimanov, Elena V. Mikhalchik, Nailya T. Urmantaeva, Emin L. Salimov, Aligeydar A. Ragimov, Tatyana M. Khlebnikova, Peter S. Timashev

**Affiliations:** 1 Institute for Regenerative Medicine, I. M. Sechenov First Moscow State Medical University, Moscow 119991, Russia; 2 Lopukhin Federal Research and Clinical Center of Physical-Chemical Medicine of Federal Medical Biological Agency, Moscow 119435, Russia; 3 Department of Blood Transfusion, I. M. Sechenov First Moscow State Medical University, Moscow 119991, Russia; 4 World-Class Research Center “Digital Biodesign and Personalized Healthcare”, I. M. Sechenov First Moscow State Medical University, Moscow 119991, Russia

**Keywords:** neutrophils, platelets, pericardium scaffolds, reactive oxygen species, chemiluminescence

## Abstract

Implantation of scaffolds causes a local inflammatory response whereby the early recruitment of neutrophils is of great importance not only for fighting the infection, but also for facilitating effective regeneration. We used luminol-dependent chemiluminescence, flow cytometry, ELISA, and confocal microscopy to assess the responses of neutrophils after the exposure to the scaffold-decellularized bovine pericardium (collagen type I) crosslinked with genipin (DBPG). We demonstrated that DBPG activated neutrophils in whole blood causing respiratory burst, myeloperoxidase (MPO) secretion, and formation of neutrophil extracellular trap-like structures (NETs). In addition, we studied platelets, another important player of the immediate immune host response. We found that platelets triggered redox-activation of isolated neutrophils by the pericardium scaffold, and likely participate in the NETs formation. Free radicals generated by neutrophils and hypochlorous acid produced by MPO are potent oxidizing agents which can oxidatively degrade biological structures. Understanding the mechanisms and consequences of redox activation of neutrophils by pericardium scaffolds is important for the development of new approaches to increase the efficiency of tissue regeneration.

## 1. Introduction

The goal of regenerative medicine is to provide a substitute for damaged or lost tissues. The tissues loss can occur not only due to trauma but in different pathologies such as cardiovascular diseases, rheumatoid arthritis, gastroenterology, congenital abnormalities, etc. One of the most efficient approaches to reconstitute the tissues is the use of biomeshes providing a niche for stimulation of tissue formation. The key elements of tissue engineering constructs are three-dimensional biocompatible degradable scaffolds capable of stimulating the regeneration of lost and damaged tissues [1,2]. The immune response to scaffold implantation is initiated by two factors: (1) damage to tissues and blood vessels during surgery causes the release of “alarm signals” (proteins of damaged matrix, intracellular proteins) and (2) the adsorption of plasma proteins (proteins of the complement system and coagulation cascade, immunoglobulins) on the surface of a material triggers the reactions of hemostatic system and development of an acute inflammation in which neutrophils and macrophages play the major role.

Neutrophils are innate immune cells recruited and activated at the site of local tissue damage earlier than other cells; they largely determine the overall response of the immune system to scaffold implantation. Research is mainly focused on neutrophils associated with scaffolds and located in the surrounding tissues. Suliman et al. [3] showed that after subcutaneous implantation of poly((L-lactide)-co(ε-caprolactone)) scaffolds in animals, the injected material was infiltrated with neutrophils and lymphocytes along the periphery of the scaffold for a week after surgery. Sadler et al. [4] compared the immune response induced by the implantation of synthetic scaffolds and the scaffold based on extracellular matrix. The authors showed that all materials induced a similar immune response, but in the case of synthetic materials, more neutrophils were recruited. The degree of immune response depended on the surface and mechanical properties of the synthetic material.

Bioprosthetic materials based on an extracellular matrix are of particular interest in medicine due to their availability, low cost, and tunability [5]. Decellularized pericardium (DP) contains a variety of structural proteins which provide mechanical strength, structural anisotropy, biocompatibility, biodegradability, etc. Decellularized bovine pericardium (DBP) is widely used in reconstructive surgery: cardiac surgery, orthopedics, ophthalmology, general and pulmonary surgery, and dentistry [6,7,8]. However, successful clinical application of the material is complicated by its immunogenicity and proteolytic degradation. DBP tissue is mainly composed of collagen and elastin embedded into the amorphous matrix consisting of glycosaminoglycans and proteoglycans. The prevailing protein component is collagen type I. A common method to reduce the inflammatory potential of scaffold and to protect collagen from biodegradation is crosslinking [8,9,10]. Glutaraldehyde (GA) is traditionally used for this purpose. GA binds tropocollagen helices with each other with formation of intra- and intermolecular bridges in the tissue. A number of alternative approaches to the pericardium tissue crosslinking have been developed. The highest proteolytic stability was found for ethylene glycol diglycidyl ether (EGDE), an epoxy compound [8]. One of the most promising crosslinkers is the naturally occurring crosslinking agent genipin. Like GA, genipin crosslinks collagen by reacting with primary amino groups and demonstrates a high crosslinking index [9]. Multipoint covalent crosslinking hinders collagen digestion due the inability of collagenase to penetrate into a biomaterial and unwind triple-helical tropocollagen regions. As in the case of any matrix-based biomaterial, implanted DP inevitably causes an immune response, the specific features of which are determined by the properties of the materials [1].

In vivo studies demonstrated a decrease in immunogenicity of biomaterials crosslinked with genipin. Genipin-crosslinking alleviated the host response to xenogeneic-decellularized porcine whole liver matrices by reducing the proliferation of lymphocytes and their subsets, accompanied by a decreased release of both Th1 and Th2 cytokines [11]. At the same time, histologic analysis of the liver xenografts demonstrated the similar infiltration by neutrophils of genipin and glutaraldehyde groups at day seven after implantation and increase in neutrophil count in GA group in the late postoperative period. In vivo evaluation of cellular and acellular bovine pericardia revealed the degrees in inflammatory reaction for the genipin-fixed tissues were significantly less and shorter than their glutaraldehyde-fixed counterparts [12]. Additionally, the tissue regeneration rate for the genipin-fixed acellular tissue was significantly faster.

Infiltration of tissue around a scaffold by neutrophils occurs several hours after surgery, whereas the first immune cells engaged with scaffold are blood neutrophils from damaged vessels. Neutrophils comprise 50–70% of all leukocytes circulating in the blood, and their concentration is 2–7 million/mL. Blood neutrophils appear at the site of implantation at the beginning of a surgery and continue to arrive until the bleeding stops. Nevertheless, to date, there have been no studies on the reduction/oxidation (redox) response of neutrophils to extracellular matrix-based scaffolds in blood.

Neutrophil activation is manifested in: (1) changes in the morphological and functional properties of cells, (2) phagocytosis of foreign intruders, (3) secretion of the contents of intracellular granules and cytokines, (4) production of reactive oxygen species (ROS) and oxidants, (5) formation of neutrophil extracellular traps (NETs) [13,14,15,16]. In our study, we characterize the redox activation of neutrophils, namely their ability to produce redox active compounds (ROS and other oxidants) which create an oxidative pro-inflammatory environment around implanted material.

Neutrophils trigger oxidative burst and bacterial killing via activation of two major enzymes—NADPH oxidase and myeloperoxidase (MPO). MPO is an enzyme of azurophilic granules that is secreted into phagosomes or extracellular space by activated neutrophils. At the same time, the assembly and activation of membrane-bound NADPH oxidase leads to the synthesis of superoxide radicals (O_2_^•−^), which dismutate to form hydrogen peroxide. In the presence of H_2_O_2_, MPO produces hypochlorous acid (HOCl)—the highly reactive oxidant which contributes to host defense against pathogens [15,17]. In addition, neutrophils form NETs, a “sticky network” consisting of intracellular granule proteins (MPO, elastase, lysozyme, etc.), DNA chromatin, and histones. MPO is active in NETs and can also synthesize hypochlorous acid (HOCl) in the presence of H_2_O_2_. ROS and HOCl produced by neutrophils have a high oxidative capacity and can oxidase not only bacteria and surrounding tissues, but also a scaffold, which can influence the further immune response of the body and wound healing.

Platelets are non-nuclear tiny blood cells participating in thrombosis. In recent years, there is increasing evidence that platelets play an important role in the processes of inflammation and tissue regeneration. The interplay of platelets and neutrophils is crucial at the early stages of inflammation, since activated platelets can stimulate neutrophil migration and activation via CD40L/CD40 and P-selectin/PSGL1 interactions [18,19]. Formation of platelet–neutrophil complexes in blood promotes the recruitment of neutrophils to inflamed tissue and NETs formation as well enhances neutrophil phagocytosis and respiratory burst. ROS and HOCl produced by activated neutrophils are very strong non-selective oxidants, they damage surrounding tissues as well as platelets and neutrophils, leading to cell death [18].

In vitro studies of cell response to implanted materials are usually performed by cell line culture. A 3D model was developed to mimic the wound niche and fibrotic tissue formation: the primary macrophages in a plasma-derived fibrin hydrogel with integrated fibroblasts were exposed to biomaterials for 13 days [20].

Using fluorescence lifetime imaging (FLIM), we demonstrated hypochlorite (NaOCl)-induced changes in fluorescence decay parameters of DBP crosslinked with genipin or EGDE [21]. The fluorescence decay parameters also correlated with different degrees of oxidation and changes in micromechanical properties of the scaffolds as assessed by atomic force microscopy. Noteworthy, both crosslinkers are very effective in protecting DBP from proteolytic degradation [8,9,22]. An increase in fluorescence lifetimes of genipin-induced crosslinks in collagen at a depth of 10 µm was found after material incubation with suspension of neutrophils under conditions simulating the inflammatory site. Enzymes secreted by neutrophils (collagenases, gelatinases, etc.) are molecules which are too bulky to penetrate into a crosslinked DBP; so, enzymatic degradation is a process of surface “erosion” [23]. Changes in the physicochemical properties of the material under the surface indicate that radicals and oxidants produced by activated neutrophils are able to penetrate into the crosslinked DBP and modify it. Redox modification of the scaffold will undoubtedly affect the further immune response to its implantation. Therefore, the study of redox activation of neutrophils by scaffold is of great importance to improve the efficiency of tissue regeneration.

The purpose of our study was to elucidate the immediate immune response of blood components to scaffolds: the whole blood was exposed to the pericardium for 1–2 h. In this work, using DBP crosslinked with genipin (DBPG), we demonstrated the redox activation of neutrophils in the blood by biomaterial—ROS generation, MPO secretion, and NETs formation. We obtained quantitative characteristics of the DBPG-induced production of oxidants and showed the important role of platelets in initiating the activation of neutrophils by DBP.

## 2. Results

### 2.1. Activation of Neutrophils by Pericardium Scaffolds in Whole Blood

The study of blood cells in whole blood samples is the most appropriate way to simulate the neutrophil response in vivo. Neutrophil activation is a multistage process: morphofunctional changes are accompanied by the release of the content of intracellular granules, cytokine secretion, and ROS production [13,14]. In our study, we compared samples of blood incubated with scaffolds and control sample incubated with PBS (Figure S1a).

**Figure 1:**
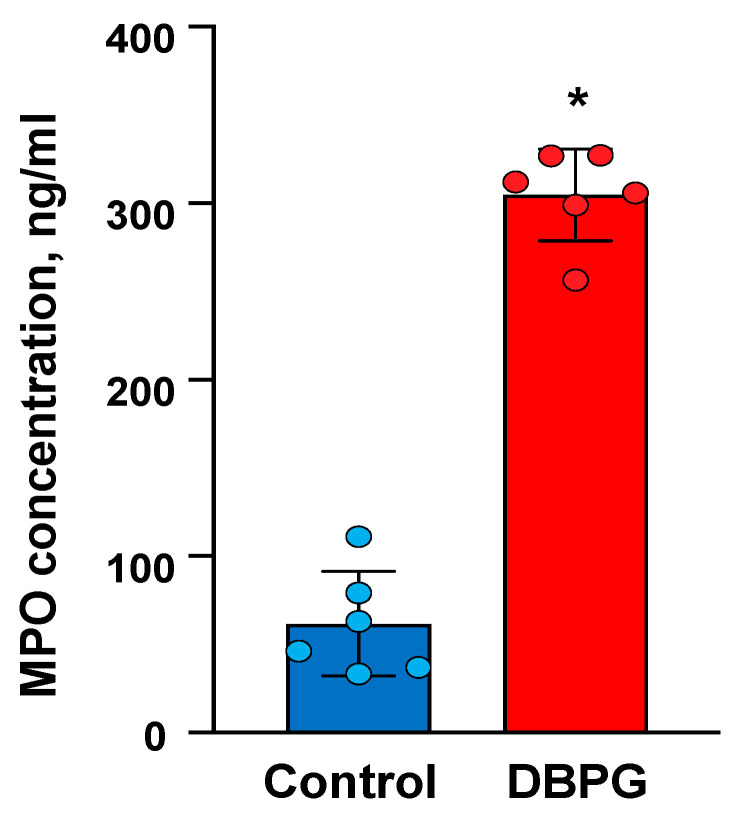
MPO concentration increased in the plasma of blood incubated with DBPG: concentration of MPO secreted by neutrophils after blood was exposed to PBS (Control) and DBPG for 1 h at 36.7 °C. Data are means ± S.D., n = 6, * *p* < 0.05 versus Control.

MPO is a specific enzyme which is secreted by activated neutrophils. We have shown a 4–5-fold increase in the concentration of MPO in plasma of blood incubated with DBPG (Figure 1). No significant difference was found between samples incubated with DBP crosslinked with different crosslinkers-genipin and ethylene glycol diglycidyl ether (DBP-EGDE) (Figure S1b).

ROS and HOCl produced by activated neutrophils are highly reactive. The simple and sensitive approaches of ROS detection utilize probes that react with radicals and oxidants with the formation of light-emitting compounds.

**Figure 2:**
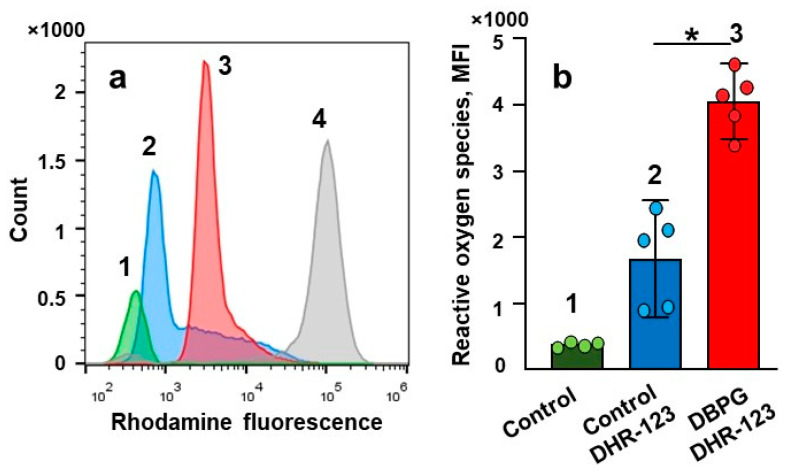
Assessment of neutrophil activation in blood by flow cytometry. Whole blood was incubated at 36.7 °C for 1 h without any addition (1), and with the addition of DHR-123 and reagents. (2, 3, 4)—blood was incubated with DHR-123 for 15 min prior to addition of PBS (2, 20 µL to 250 µL of blood), DBPG (3, 2 mg of scaffold to 250 µL blood) and PMA (4, 150 nM). (**a**) Representative histograms of DHR-123 fluorescence in neutrophils. (**b**) Level of ROS produced in neutrophils after blood incubation without any additions (1, Control) and with DHR-123 and PBS (2, Control DHR-123) or DBPG (3, DBPG DHR-123). ROS production is reported as median fluorescence intensity (MFI). Data are means ± S.D., n = 5, * *p* < 0.05 versus Control-DHR123.

First, we used dihydrorhodamine 123 (DHR-123) and flow cytometry to detect intracellular ROS production. DHR-123 is oxidized by intracellular ROS to the fluorescent compound rhodamine [24]. We have shown that incubation of whole blood with DBPG resulted in a significant increase in the fluorescence of rhodamine in the granulocyte population (Figure 2a): the median fluorescence intensity (MFI) increased more than twice (Figure 2b). The fluorescence of neutrophils in blood treated with the neutrophil activator phorbol 12-myristate 13-acetate (PMA) (150 nM) was used as a positive control.

**Figure 3:**
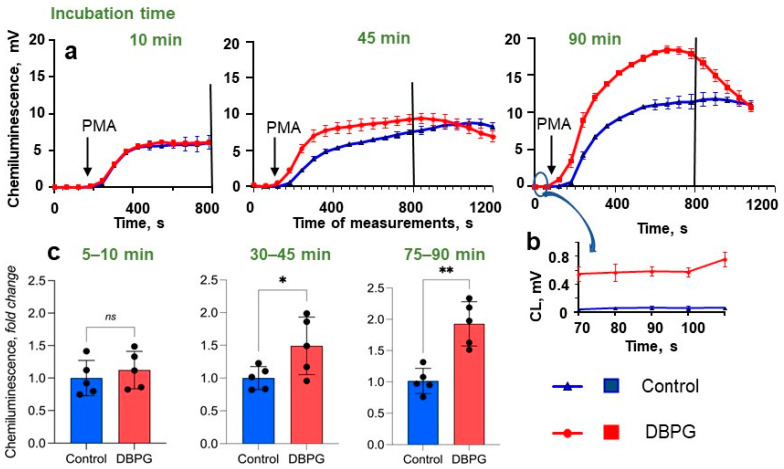
Chemiluminescence of blood samples. Blood was incubated with PBS (Control) or with DBPG at 36.7 °C. The incubation time is indicated above the CL curves (10 min, 45 min, 90 min) and the bars (green color). (**a**) Kinetics of blood CL. Blood was added at point time 0 min to the chemiluminometer cuvette containing 485 µL of Krebs–Ringer medium (with NaHCO_3_ and CaCl_2_) and 200 µM luminol. The *x*-axis of the graphs represents the time of CL measurement. The arrows indicate the addition of 100 nM PMA. Measurements were carried out at 37 °C until the CL maximum was reached for all samples. Each CL curve is the average of three curves obtained by measuring of three independent probes taken from one sample (the error did not exceed 15%). (**b**) Chemiluminescence of the samples prior to PMA addition. (**c**) Levels of ROS generated by neutrophils in blood samples incubated with PBS (Control) and scaffold (DBPG). The black vertical line in CL kinetics (**a**) indicates the cutoff time of measurement (800 s) to calculate the area under CL curves. (**c**) Level of ROS (area under chemiluminescence curves) generated by neutrophils in blood samples incubated with PBS (Control) and scaffolds (DBPG) at different incubation times (fold change relative Control). The incubation time (green) is indicated above bars. Data are means ± S.D., n = 5. * *p* < 0.05 vs. Control, ** *p* < 0.01 vs. Control, ^ns^ *p* > 0.05 vs. Control.

Next, luminol-dependent chemiluminescence (CL) was employed to monitor the neutrophil activation. Luminol-dependent CL is a fast, simple, and sensitive method for assessment of the level of neutrophil activation in whole blood [16,25]. Luminol is oxidized by ROS and after a number of intramolecular rearrangements, it goes over 3-aminophthalate, which is accompanied by the emission of a quantum of light. We took aliquots of blood incubated with PBS (Control) or with scaffold at different incubation times and added them into the cuvette of chemiluminometer containing Krebs–Ringer buffer and luminol (Figure 3a). No significant differences in CL without inductor or with PMA were detected in 10 min of incubation. However, after 45 min of incubation, neutrophils became activated in the sample with scaffold compared to control; they generated ROS faster in response to PMA, i.e., samples incubated with DBPG reached the CL peak earlier than control samples which evidences neutrophil priming in these samples. As the incubation time increased to 90 min, the differences between the samples increased. At longer incubation times, a higher level of CL measured before PMA addition was observed in the sample with DBPG, which indicated scaffold-induced activation of neutrophils (Figure 3b). The area under a CL curve is proportional to the amount of ROS produced. The time-dependent increase in the difference between the areas under CL curves means scaffold-induced activation of neutrophils in the blood to produce ROS (Figure 3c). Similar results were obtained for DBP-EGDE.

CL curves of control samples measured after PMA addition to blood varied significantly in shape and amplitude from donor to donor (Figure 3, Figures S2 and S3). Erythrocytes and hemoglobin may affect the kinetics of blood CL [26]. The variation may be due to a different number of neutrophils and red blood cells in the blood of different donors, partial lysis of red blood cells, etc. At the same time, a good reproducibility of CL curves was observed within one experiment for two or three replicates at a given incubation time (Figures S2 and S3). Comparison of the areas under the CL curves allowed us to exclude the difference between the donors from consideration and conclude that incubation of whole blood with DBPG caused an increase in CL response of blood to PMA compared to the control: the peak time of CL was shorter and/or CL amplitude increased, and consequently, the area under the CL curve increased what evidenced the augmentation in ROS generation (Figure 3c, Figures S2c and S3c).

Treatment of the scaffold with autologous plasma before exposure to blood did not affect the scaffold-induced neutrophil activation in whole blood (Figure S2). In separate experiments, we used the antibiotic polymyxin B sulfate, which is a well-known agent for the removal of endotoxins. DBPG was treated with a solution of polymyxin B (100 µM in PBS) for 20 min, and then, it was placed into the blood containing 20 µM of antibiotic. Polymyxin B did not change significantly the activation capacity of DBPG (Figure S3).

The oxidation of luminol is nonspecific and can be achieved by interaction of the molecule with various radicals and oxidants. Both ascorbate (scavenger of ROS) and methionine (scavenger of HOCl) decreased luminol oxidation by activated neutrophils in blood which evidences the participation of free radicals and HOCl in luminol oxidation (Figure S4).

We used simple and fast CL method to test neutrophil activation by clinically used absorbable wound coverage “KOLLAGEN resorb” (RESORBA Medical GmbH, Nürnberg, Germany) (Figure S5). This sponge is a very light material, we added it to the blood at a ratio of 1 mg to 250 µL of blood and observed a significant activation of neutrophils during long-term incubations (50 min or more). A very inhomogeneous material surface caused a large experimental error.

Blood containing low levels of erythrocytes was incubated with DBPG and then incubated with DAPI. Confocal microscopy images revealed large DNA-containing structures which were larger than leukocytes (>15 µm) and looked like NETs (Figure S6).

Thus, experiments with whole blood showed that DBP activated neutrophils, amplifying the MPO release and ROS production.

### 2.2. Activation of Isolated Neutrophils by Pericardium Scaffold

**Figure 4:**
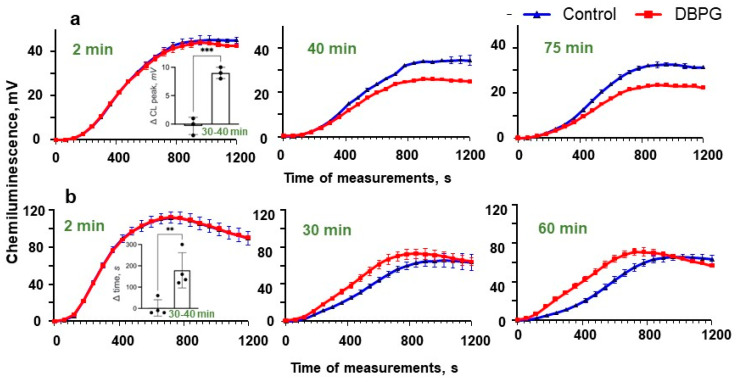
CL of isolated neutrophils (2 million cells/mL) resuspended in plasma (**a**) and in platelet-rich plasma (PRP) (**b**). Autologous plasma accounted for 70% of the total solution volume. The blue curves present the CL kinetics for the control sample, the red curves show the CL of a sample incubated with DBPG (1 mg of scaffold per 125 µL of neutrophil suspension) at 36.7 °C. The incubation time is indicated above the CL curves. Blood was added at point time 0 min to the chemiluminometer cuvette containing 485 µL of Krebs–Ringer medium (with NaHCO_3_ and CaCl_2_) and 200 µM luminol, then 100 nM PMA was added. The *x*-axis of the graphs represents the time of CL measurement. Measurements were carried out at 37 °C until the CL maximum was reached for all samples. Each CL curve is the average of three curves obtained by measuring of three independent probes taken from one sample (the error did not exceed 15%). Inserts show comparison of CL parameters for the experiments with PPP (difference in CL maximum) and PRP (difference in CL peak time) after 2–5 min and 30–40 min of incubation. Further incubation did not significantly change the ratio of parameters. Data are means ± S.D., n = 3 and 4, ** *p* < 0.01 vs. Control, *** *p* < 0.001 vs. Control.

To study the mechanism of DBPG-induced neutrophil activation, we compared DBPG-activation of neutrophils in plasma and in platelet rich plasma (PRP) using CL (Figure 4). If plasma was present in the suspension of isolated neutrophils, the CL peak of a PMA-stimulated neutrophils was lower in the samples preincubated with DBPG vs. control. The decrease in the CL intensity of neutrophil suspension may be due to neutrophil adhesion onto the material. In our experiments, a 15% decrease in the number of cells was observed after the neutrophil suspension was incubated with the scaffold (0.25 million cells per mL) for an hour at 36.7 °C (Figure S7c).

When neutrophils were incubated in the presence of PRP, a decrease in CL amplitude was not observed, but the time of CL peak of the sample incubated with DBPG was shorter and, consequently, the area under the CL curve increased, which indicated neutrophil activation by the material (Figure S7a,b). The effect was time-dependent. These results suggest that platelets are active players in DBP-induced neutrophil activation in whole blood.

### 2.3. Platelet Activation in Blood Exposed to DBPG

Platelets play an important role in the inflammatory process by interrelating with other cells of the immune system. Platelets are activated at the site of tissue damage earlier than any other blood cells and secrete cytokines that promote the migration of neutrophils to the site of injury [19,27].

**Figure 5:**
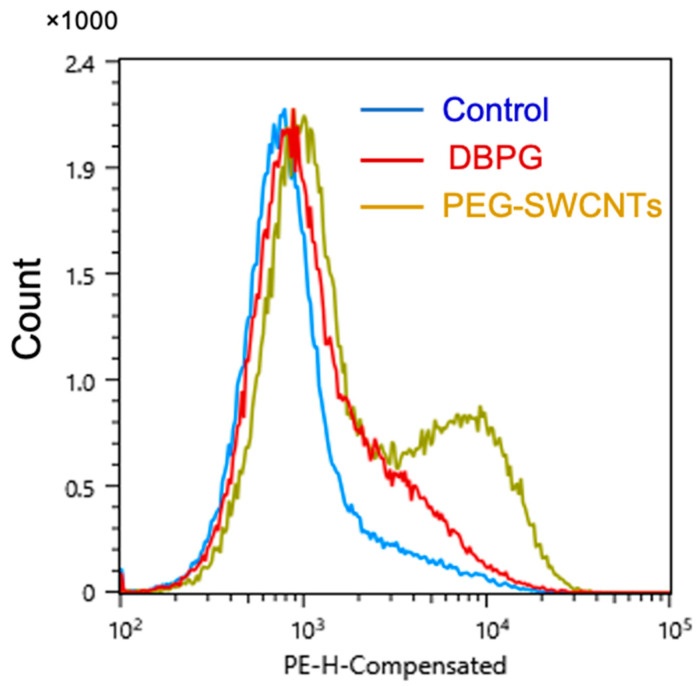
Flow cytometry of platelet rich plasma. PRP was obtained by centrifugation (150 g) after blood incubation with DBPG or pegylated single-walled carbon nanotubes (PEG-SWCNTs, 75 µg/mL in blood) at 36.7 °C for 45 min. Control sample was incubated with 20 µL PBS. Platelet activation marker P-selectin was detected by anti-CD62P-PE antibodies. The results are typical of three independent experiments.

Platelet activation is accompanied by the expression of P-selectin on their surface. We used anti-CD62P antibodies labeled with phycoerythrin (PE) to detect activated platelets in PRP by flow cytometry. DBPG-induced activation of single platelets was significant compared to the control but very low, much less than that induced by pegylated single-walled carbon nanotubes (PEG-SWCNTs), which, as shown earlier, activate platelets in the blood [28] (Figure 5). Flow cytometry detects individual cells or small aggregates of platelets in the control sample. However, platelets can form large aggregates, including those initiated by platelet interaction with NETs [29].

**Figure 6:**
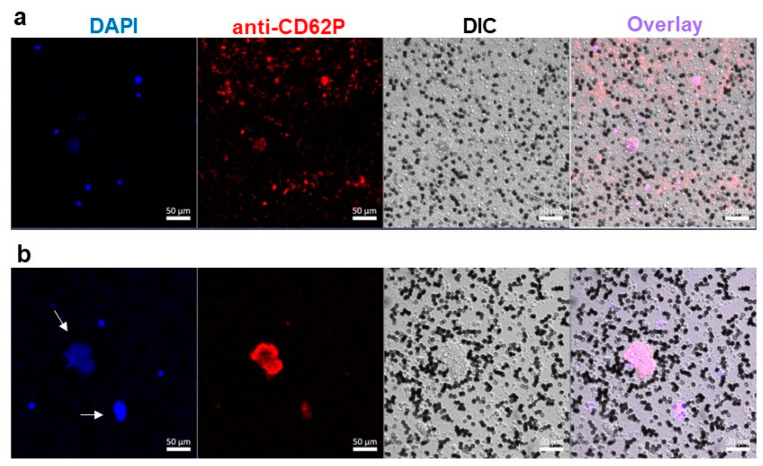
Confocal microscopy of erythrocyte-poor blood samples incubated without addition (**a**) or with DBPG (**b**). DAPI—nuclear staining; anti-CD62P-PE—activated platelet staining. Black cells are erythrocytes. Arrows indicate accumulation of chromatin larger than 15 µm. DIC—differential interference contrast.

Confocal microscopy was employed to simultaneously follow neutrophil activation and platelet aggregate formation in blood with a low content of erythrocytes (erythrocyte-poor blood, ErPB). In the control sample, DAPI-staining revealed individual nucleated cells (Figure 6a). These cells are living leukocytes, since the incubation with a relatively high concentration of DAPI was long, the dye could penetrate into the cell. Bright field imaging has shown multiple small cells covering the entire field, they are settled platelets. Some platelets form small aggregates of 3–5 cells (less than 15 µm in diameter) in which they are activated (anti-CD62P-PE stain). In an ErPB sample incubated with DBPG, large chromatin areas with a size of more than 15 μm (the size of leukocyte) were observed, which looked like NETs (Figure 6b and Figure S6). The number of single neutrophils and platelets decreased, at the same time, very large platelet aggregates appeared, which could be detected separately or colocalized with NETs.

## 3. Discussion

Scaffolds promote the regeneration of lost and damaged tissues, they stimulate cell recruitment and tissue remodeling, and, at the same time, they are gradually degraded. A coordinated immune response to a scaffold determines both the rate of scaffold degradation and the effectiveness of wound healing. Neutrophils and macrophages are phagocyting cells that play a crucial role in wound healing. Neutrophils are present at the site of implantation during the first week after surgery. Macrophages are the major performers and conductors of regenerative processes at all stages of the inflammatory response. Many studies are devoted to the functions of immune cells, and primarily to the activation of macrophages into the classically activated proinflammatory M1 phenotype or alternatively activated reparative anti-inflammatory M2 phenotype. Different approaches were suggested to optimize macrophages functions [2,30,31,32]. At the same, little attention has been paid to the role of neutrophils in the driving of the immune response.

The development of acute inflammation occurs as a result of activation of the innate immune system. Neutrophils are the first immune cells to arrive at the site of surgery, and their major role is not only to fight the infection but also to modify a damaged tissue to ensure effective regeneration. Neutrophils secrete proteins, such as collagenase and gelatinase, digest the extracellular matrix, which results in the formation of “tunnels” for large macrophages and further angiogenesis. At the same time, these enzymes cause modification of extracellular matrix-based scaffolds. Digestion of bovine pericardium collagen by collagenase was demonstrated by fluorescence lifetime imaging [33].

However, to date, little is known about the role of oxidative stress mediated by neutrophil activation in tissue regeneration. Upon neutrophil activation, NADPH oxidase generates superoxide anions on both sides of the plasma membrane which dismutate to form hydrogen peroxide. H_2_O_2_ is a fuel for MPO active site to produce HOCl. O_2_^•−^, H_2_O_2_ (in the presence of iron ions), and HOCl are redox-active compounds capable to modify tissues and scaffold material which later can have a significant impact on the polarization of macrophages and the efficiency of tissue healing.

In our work, reduction/oxidation (redox) activation of neutrophils exposed to DBP was demonstrated in whole blood. This approach allowed us to study the specific response of blood cells to a scaffold and not to tissue damage during surgery. DBP crosslinked with genipin was studied because fixation of biomaterials with genipin reduced significantly their immunogenicity and biodegradability as demonstrated in in vivo experiments [11,12]. Some of the measurements were made with DBP crosslinked with ethylene glycol diglycidyl ether [8].

We employed flow cytometry and CL to detect ROS and oxidants produced by neutrophils in blood exposed to DBPG. Flow cytometry revealed an increase in intracellular ROS generation (Figure 2). During the oxidative burst, intracellular DHR-123 is converted to rhodamine through oxidation mainly by H_2_O_2_ [13,24]. Luminol-enhanced CL is one of the most informative techniques to study both extra- and intracellular generation of ROS, namely O_2_^•−^ and HOCl, by activated neutrophils [16,34]. Additionally, luminol is a peroxidase substrate which can be oxidized by secreted MPO in the presence of H_2_O_2_ [35]. Incubation of blood with DBP resulted in time-dependent amplification of neutrophil activation by PMA as demonstrated by luminol-dependent CL (Figure 3, Figures S2 and S3). An increase in the response of cells to the activator PMA evidences the neutrophil priming with the scaffold. The simple and fast CL method can be used to test the ability of any biomaterial to prime the neutrophils activation in whole blood. We showed neutrophil-activation propensity of absorbable wound coverage “KOLLAGEN resorb” (RESORBA Medical GmbH, Nürnberg, Germany) (Figure S5).

An elevated amount of MPO in the plasma of blood incubated with the scaffold proved the degranulation of neutrophils (Figure 1). In blood samples incubated with a scaffold, confocal microscopy revealed large extracellular DNA complexes which are similar to NETs and can contain neutrophil granule proteins including MPO (Figure S6) [14,15]. In the presence of H_2_O_2_, both MPO in plasma and MPO in NETs are active to produce hypochlorous acid.

ROS generated by activated neutrophils not only have a bactericidal function but promote signal transduction by modifying the redox state of proteins and lipids [36,37]. Extracellular ROS production and the ability of H_2_O_2_ to diffuse over 100 µm in the extracellular space enable paracrine ROS signaling outside neutrophils and in neighboring cells [38]. For example, protein kinase C activity can be modulated by H_2_O_2_ through oxidation of cysteins in regulatory and catalytic domains [39]. HOCl produced by MPO activates latent matrix metalloproteases by covalently modifying the free thiol of the prodomain cysteine residue [40]. ROS produced by neutrophils were found to be important mediators that trigger the phenotypic conversion of macrophages to pro-resolving state-orchestrating liver repair [41].

Free radicals and HOCl generated by activated neutrophils and macrophages were shown to be able to oxidize and degrade various materials, including rigid and chemically stable structures such as carbon nanotubes causing changes of their structure and properties [42,43]. One may expect oxidative degradation of implanted materials (Figure S8). In vivo degradation of polyetherurethane elastomers by both hypochlorous acid and nitric oxide-based oxidants was demonstrated by attenuated total reflectance Fourier transform infrared spectroscopy [44]. Using FLIM, the high sensitivity of fluorescence lifetime of genipin crosslinks to redox modification led us to detect the oxidation of DBPG after its incubation with isolated neutrophils at the experimental conditions simulating the inflammatory site [21]. The changes in material fluorescence decay parameters were detected at the depth of 10 µm suggesting that modification was caused by oxidative reactions. Subsequent chemical/biochemical changes occurring in the material after treatment with NaOCl were accompanied by sample bleaching (Figure S9).

An oxidative microenvironment created by neutrophils and redox modification of the biomaterial surface by neutrophil-derived oxidants are expected to affect the further activity and functions of macrophages and scaffold degradation [45]. In addition, one should take into account the possible oxidation of various substances associated with implants: implantable drug-delivery systems, scaffold-bound molecules such as cytokines and others which are used for scaffold functionalization to regulate the immune response [30,32,46,47].

Neutrophil activation and NaOCl-induced scaffold degradation did not depend substantially on the crosslinking agent used for the preparation of pericardium-derived scaffolds. Release of MPO by activated neutrophils was similar when the pericardium was modified by the natural crosslinking agent genepin or chemical epoxy compound (Figure S1b). The degradation of DBP crosslinked with ethylene glycol diglycidyl ether was detected in the presence of NaOCl by FLIM [21].

To study the mechanisms of neutrophil activation by the pericardium, we performed experiments with isolated neutrophils in plasma (Figure 4). The CL intensity of PMA activated cells decreased if the cells were incubated with DBPG in the presence of plasma, which can be explained by the binding of some cells to the surface of the material and cell death or damage. When neutrophils were incubated with the scaffold in the presence of platelet-rich plasma, the decrease in CL intensity was not so pronounced (if any), instead the shortening of the CL peak time was observed, which proved the involvement of platelets in the redox activation of neutrophils by the scaffold in blood.

Not only proteins of the immune system associated with a material but also platelets activated on its surface stimulate the activation of neutrophils [1,48]. Platelets are best known as key players in thrombosis and hemostasis; recent data demonstrate the important immune and inflammatory roles of platelets in both health and disease. Experimental and clinical evidences for the role of platelet–neutrophil crosstalk in tissue injury or repair and in the pathogenesis of inflammatory diseases such as transplant rejection, atherosclerosis, rheumatoid arthritis, etc., are rapidly emerging [27,29,49,50]. In early puerperium, circulatory neutrophils exhibit enhanced neutrophil extracellular trap formation which was detected as elevated levels of circulating DNA in serum. Release of NETs enriched by the tissue factor near damaged endothelium caused alterations in the platelet activity status and activation of the coagulation cascade [50].

Upon activation, platelets express an array of surface immune receptors and adhesion molecules and release various chemokines and cytokines with immunomodulatory functions to regulate leukocyte migration, phagocytosis, and ROS generation. Platelets are a major source of proinflammatory IL-1ß which has been identified as an important neutrophil activator and pro-survival cytokine [27]. Because of these characteristics, and their high numbers in the circulation (150–400 million platelets/mL), platelets are able to modulate the host immune response [19].

Exposure of biomaterials to blood causes not only protein adsorption but platelet adhesion, blood coagulation, and NET formation on the material surface [51]. All these processes are relatively fast (minutes) and depend on the surface properties: chemical structure, topography, and roughness [52]. Electron scanning microscopy was employed to demonstrate platelet adsorption on the pericardium fixed with glutaraldehyde or genipin [6,53]. The adsorbed platelets and NETs on the material surface release cytokines and cell content which induce activation of platelets and neutrophils in blood not in contact with a material.

In our experiments, the number of single platelets in the sample incubated with DBPG decreased and they are slightly activated compared to the control and to blood exposed to PEG-SWCNTs (Figure 5 and Figure 6) [28]. Most platelets are activated in large aggregates which can colocalize with NETs. Binding of activated platelets to the surface of neutrophils may enhance the formation of NETs [19,29].

The actions of platelets in the inflammatory response are context-dependent. Under certain experimental conditions, platelets downregulate the oxidative burst, whereas in others, platelets contribute to adverse inflammatory outcomes. Activated platelets were demonstrated to initiate or amplify various neutrophil functions including phagocytosis, production of reactive oxygen species, MPO release, and production of NETs [27,29]. Our study demonstrated that platelets contribute to activation of neutrophils in blood by pericardium scaffolds.

Appropriate modification of a material surface to reduce neutrophil activation and platelet adhesion can allow to attenuate the neutrophil redox response [52,54]. The study of mechanisms of neutrophil activation at the site of implantation is important for the correct biomaterials’ functionalization to modulate the immune response and improve tissue repair.

## 4. Materials and Methods

### 4.1. Sample Preparation

In accordance with protocols approved by the Ministry of Health of the Russian Federation, venous blood was collected from healthy volunteers in blue top vacutainers with 3.8% sodium citrate as anticoagulant. All donors have signed an informed consent form approved by the local ethics committee of Sechenov University (No, 07–17, 13 September 2017, Moscow, Russia). The number of neutrophils in the blood was in the normal range of neutrophil count for healthy donors: 2–5 million cells per mL. Blood was subjected to centrifugation at 150 g to obtain PRP or at 250 g to obtain plasma (10 min each). To remove all erythrocytes, the collected fractions were centrifuged again.

Human neutrophils were isolated with Histopaque 1.077 (Sigma, St. Louis, MO, USA) as described in [55]. Human blood was mixed with 6% dextran T-500 (Sigma, St. Louis, MO, USA) in PBS at the 5:1 ratio, and erythrocytes were allowed to sediment for 30 min at room temperature (RT). The leukocyte-rich plasma (3–5 mL) was layered on top of 3 mL Histopaque 1.077 and subjected to centrifugation for 30 min at 400 g (RT). Erythrocytes were removed by hypotonic lysis with cold H_2_O. Neutrophils were washed twice with PBS (4 °C); the isolated neutrophils were suspended in Hanks’ Balanced Salt Solution without Ca^2+^ and Mg^2+^ at concentration of 15–17 million cells per mL. Before an experiment, neutrophils were diluted with plasma at concentration of 2 million per mL and Ca^2+^ and Mg^2+^ were added at concentration of 1 mM and 0.5 mM, respectively.

DBP was produced as described earlier (9), sterilized with gamma radiation, and prepared for the experiments under sterile conditions. Pieces of DBPG (2.5–5 mg) were placed into 10–15 mL water (water was changed 2 times) for 24 h, then for 2–3 h into 5 mL PBS. In the experiments, a piece of scaffold was put into blood or suspension of neutrophils (scaffold:suspension = 1 mg to 120 μL). A total of 20–30 µL PBS was added to blood or cell suspensions to prepare the control samples (Figure S1). Samples were incubated at 36.7 °C with gentle pipetting every 20 min. At different time intervals, cell suspensions were taken for measurements.

Absorbable wound coverage “KOLLAGEN resorb” was purchased from RESORBA Medical GmbH (Nuremberg, Germany). Pieces of sponge (1.5–2 mg) were prepared for the experiment in the same way as described for the scaffold. A piece of sponge was placed in the blood at a ratio of 1 mg to 250 µL and incubated at 37 °C with gentle pipetting every 20 min.

### 4.2. Myeloperoxidase Measurements

After incubation, cells were spun down at 250 g for 10 min. The MPO secreted by activated neutrophils was measured in supernatant using standard kits according to the manufacturer’s protocol (MPO Human Instant ELISA™ Kit, Thermo Fisher Scientific, Waltham, MA, USA).

### 4.3. Flow Cytometry

Intracellular ROS generated by neutrophils were determined by flow cytometry, which is a sensitive and highly specific method to study the ROS production in phagocytes. Fluorogenic dye DHR123 was added to the blood at a concentration of 5 μM. After 15 min of incubation, blood samples were exposed to the scaffold for 60 min. Blood exposed to 20–30 µL PBS served as a control. PMA at a concentration of 150 nM was used as a positive control. After incubation, samples were treated with the erythrocyte lysing solution VersaLyse (Beckman Coulter, Brea, CA, USA) for 10 min at 25 °C. Then, the cells were sedimented by centrifugation (250 g, 7 min). Supernatants were removed and cell pellets were resuspended in PBS for flow cytometer analysis.

Platelet activation was assessed after incubation of blood with DBPG for 45 min at 36.7 °C. The samples were centrifuged at 1000 rpm for 10 min to obtain PRP. A total of 100 µL PRP was incubated with 5 µL antiCD62P antibodies conjugated with phycoerythrin for 30 min. SWCNTs covalently functionalized by 600 Da PEG were purchased from Carbon Solutions Inc. (Riverside, CA, USA). PEG-SWCNT suspensions were prepared in deionized water by sonication for several hours at temperatures not higher than 10 °C in an ultrasonic bath (Elma Ultrasonic, Singen, Germany). The supernatant was collected after centrifugation at 12,000× *g* for 30 min and kept as a stock (1–2 mg SWCNTs/mL) in the dark at 4 °C.

Fluorescence intensity was measured using a cell sorter Sony SH-800 (Sony Biotechnology, Tokyo, Japan) with 488 nm laser. For each sample, a minimum of 10,000 events were recorded. The blood leukocyte or platelet subpopulations were separated via FSC-A/SSC-A gating, using the Sony Biotechnology software for flow cytometry data visualization and analysis.

### 4.4. Luminol-Dependent Chemiluminescence

As a result of the oxidation of luminol by ROS, a quantum of light is emitted [34,35]. We used luminol-dependent chemiluminescence to characterize the response of neutrophils to scaffolds in whole blood and in a suspension of isolated neutrophils. Aliquots of 20 µL from the control sample and from the sample incubated with scaffold were added in triplicate into a 485 µL Krebs–Ringer solution containing 200 µM luminol in polypropylene cuvettes in a Lum1200 luminometer (DISoft, Moscow, Russia). CL was registered for 1–2 min, then PMA was added at a concentration of 100 nM. The chemiluminescence of the six probes was registered simultaneously at 37 °C under mild shaking. The CL kinetics were analyzed by comparing the CL peak time, CL amplitude (peak intensity), and area under CL curves.

### 4.5. Confocal Microscopy

For confocal microscopy, ErPB was sampled over settled erythrocytes after 45 min of blood storage at RT. ErPB was incubated with scaffold at 36.7 °C for 40 min (1 mg scaffold per 110–120 µL of blood). The control sample was incubated under the same conditions with PBS (10 µL to 120 µL ErPB). At the end of the incubation, the scaffold was taken out, and the chromatin marker DAPI (500 nM) was added to the sample. Incubation was continued for another 20 min at 36.7 °C. Then, 5 µL of the anti-CD62P-PE solution was added to 100 µL of the suspension. Samples were placed in confocal dishes, waited for cell sedimentation, and proceeded to obtain images. The experiments were performed on a Zeiss LSM 880 confocal microscope (Jena, Germany). The excitation wavelengths were 405 nm and 488 nm.

### 4.6. Statistics

The results are shown as mean ± SD of at least three independent experiments. Data sets were compared using unpaired Student’s *t*-test. All *p*-values were two-tailed. A *p*  <  0.05 was considered statistically significant for all experiments.

## 5. Conclusions

In our study, we quantified the reduction/oxidation (redox) activation of neutrophils in blood caused by pericardium scaffolds by measuring ROS production and secretion of the neutrophil-specific enzyme myeloperoxidase which are markers of activated neutrophils. Formation of neutrophil extracellular trap-like structures in the blood was demonstrated. Platelets appear to drive the activation of neutrophils by the scaffold and participate in NETs formation. We proposed a simple approach to determine the impact of a scaffold on neutrophils in blood ex vivo, which allows to simulate the in vivo response of blood cells to a material and exclude the effects of alarmins released by damaged tissue. The study of the mechanisms of neutrophil activation at the site of implantation can help to control the immune response and significantly improve tissue repair.

## Data Availability

The data used in the study to support the main findings will be available from the corresponding author upon request.

## Supplementary Materials

The following supporting information can be downloaded at: https://www.mdpi.com/article/10.3390/ijms232415468/s1.

## Abbreviations

CL	luminol-dependent chemiluminescence
DP	decellularized pericardium
DBP	decellularized bovine pericardium (collagen type I)
DBPG DBP	crosslinked with genipin
DBP-EGDE DBPG	crosslinked with ethylene glycol diglycidyl ether
DHR123	dihydrorhodamine 123
DIC	differential interference contrast
ErPB	erythrocyte-poor blood
FLIM	fluorescence lifetime imaging
MFI	median fluorescence intensity
MPO	myeloperoxidase
NET	neutrophil extracellular traps
PE	phycoerythrin
PEG-SWCNTs	pegylated single-walled carbon nanotubes
PMA	phorbol 12-myristate 13-acetate
PRP	platelet rich plasma
ROS	reactive oxygen species
RT	room temperature
