# Supplementary material for: Redox-Activation of Neutrophils Induced by Pericardium Scaffolds
Source: Int J Mol Sci. 2022 Dec 7;23(24):15468. doi: 10.3390/ijms232415468 (PMC9779008; doi:10.3390/ijms232415468)
Supplement: Supplementary file 1 [file ijms-23-15468-s001.zip › ijms-2014273-supplementary.pdf]

Supplementary Material for

Redox-activation of neutrophils by pericardium scaffolds

Vlasova I.I., Suleimanov Sh.K., Mikhalchik E.V., Urmantaeva N.T., Salimov E.L., Ragimov A.A., Khlebnikova T.M., Timashev P.S.

I.M. Sechenov First Moscow State Medical University (Sechenov University), Moscow, Russia
Federal Research Clinical Center for Physical Chemical Medicine, Moscow, 119435 Russia

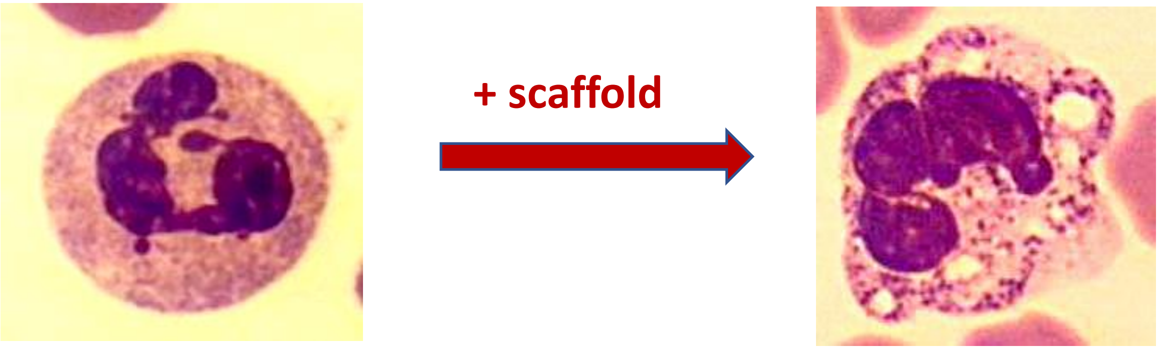

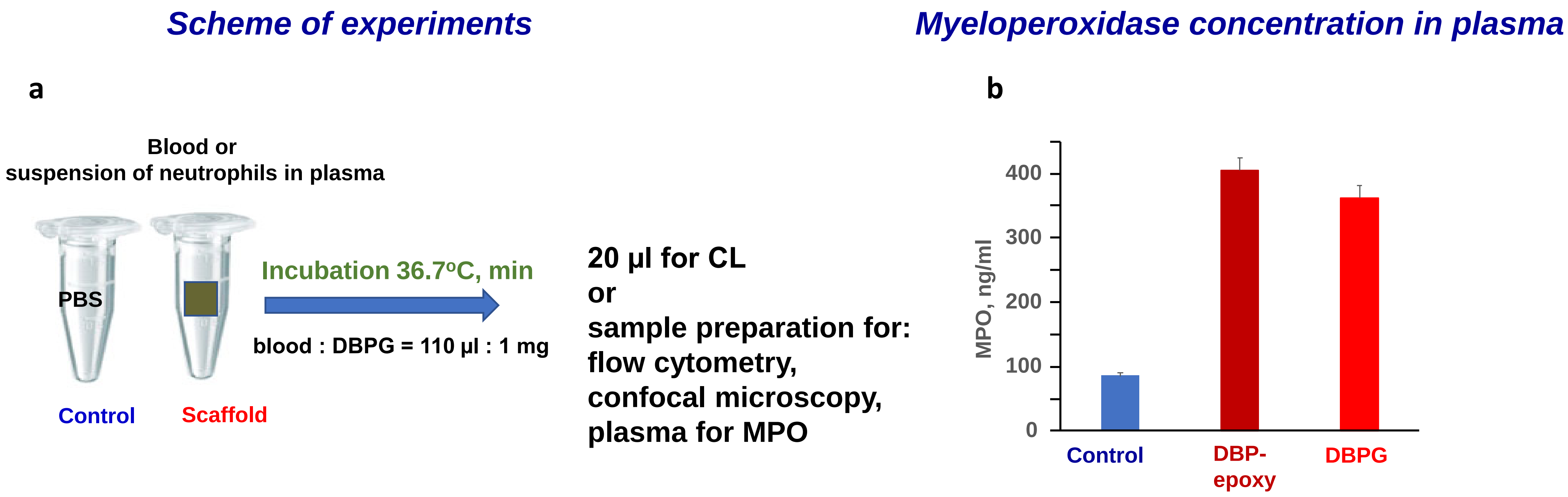

Figure S1. (**a**) **Scheme of experiments.** Details in Method Section

(**b**) **Concentration of MPO** in plasma of blood incubated with pericardium scaffolds for 1 h at 36.7C.
DBP-epoxy - DBP scaffolds cross-linked with ethylene glycol diglycidyl ether
DBPG – DBP scaffold cross-linked with genipin.

Chemiluminescence response of blood after incubation with DBPG untreated or treated with plasma

Control DBPG
Scaffold treated with plasma

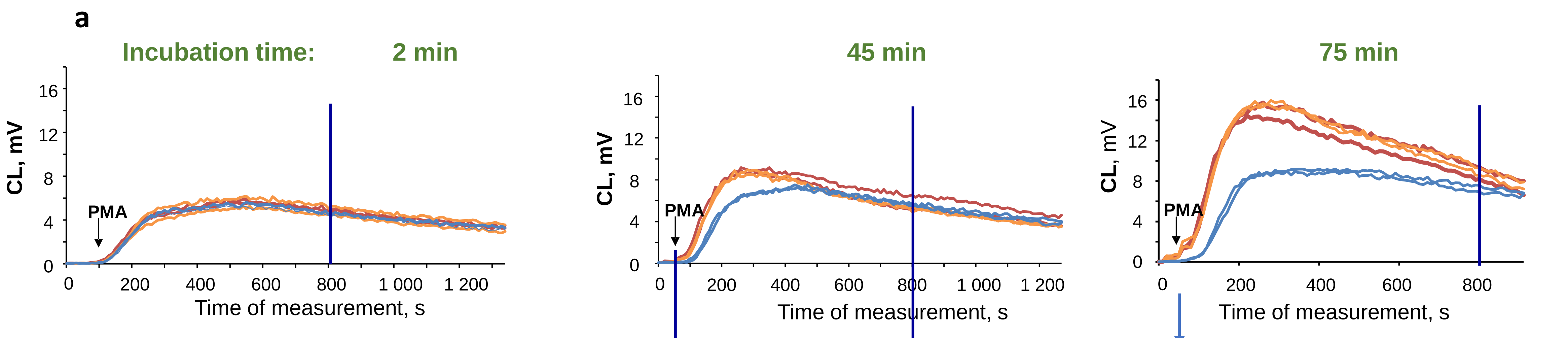

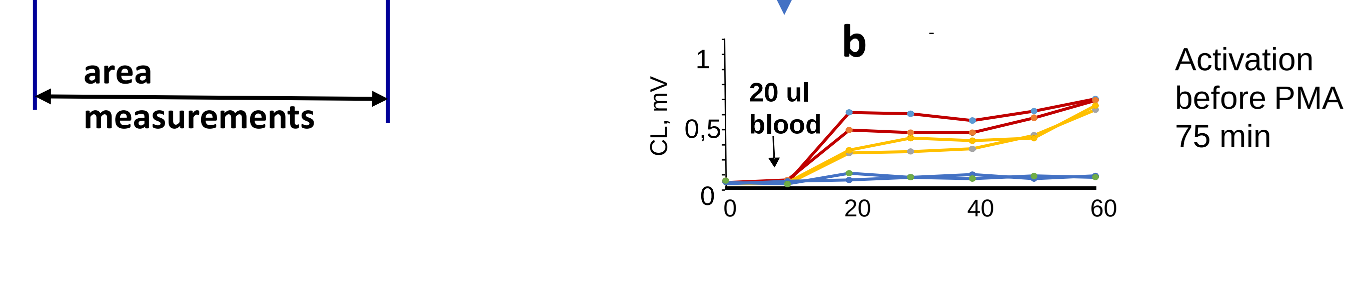

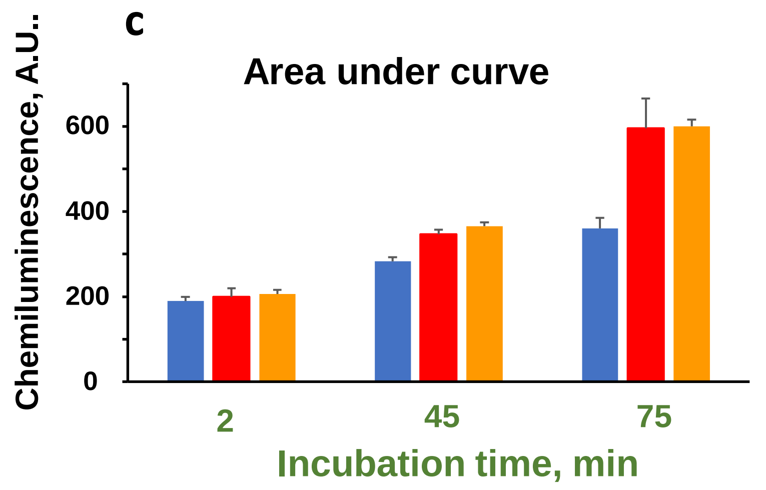

Figure S2. ROS generation by activated neutrophils in blood after incubation with DBPG untreated or treated with plasma. (**a**) Chemiluminescence (CL) of blood samples incubated at 36.7°C with PBS (Control, 20 µl), with DBPG (Scaffold) or with DBPG treated with autologous plasma (15 min 36.7° C). The incubation time (green) is indicated above the CL curves. At the end of incubation, 20 µl of blood was added to the cuvette of chemiluminometer containing 470 µl of Krebs-Ringer medium (with $NaHCO_3$ and $CaCl_2$) and 200 µM luminol. The arrows indicate the time of addition of 100 nM PMA. Measurements for a given incubation time were carried out at 37°C until the maximum was passed by all CL curves. The black vertical line indicates the cutoff time of measurements (800 s) to calculate the area under CL curves. (**b**) CL of blood after 75 min incubation with scaffold before addition of PMA. (**c**) Areas under the CL curves measured at different incubation time (indicated below the bars, green color).

Chemiluminescence response of blood after incubation with DBPG untreated and treated with polymyxin B

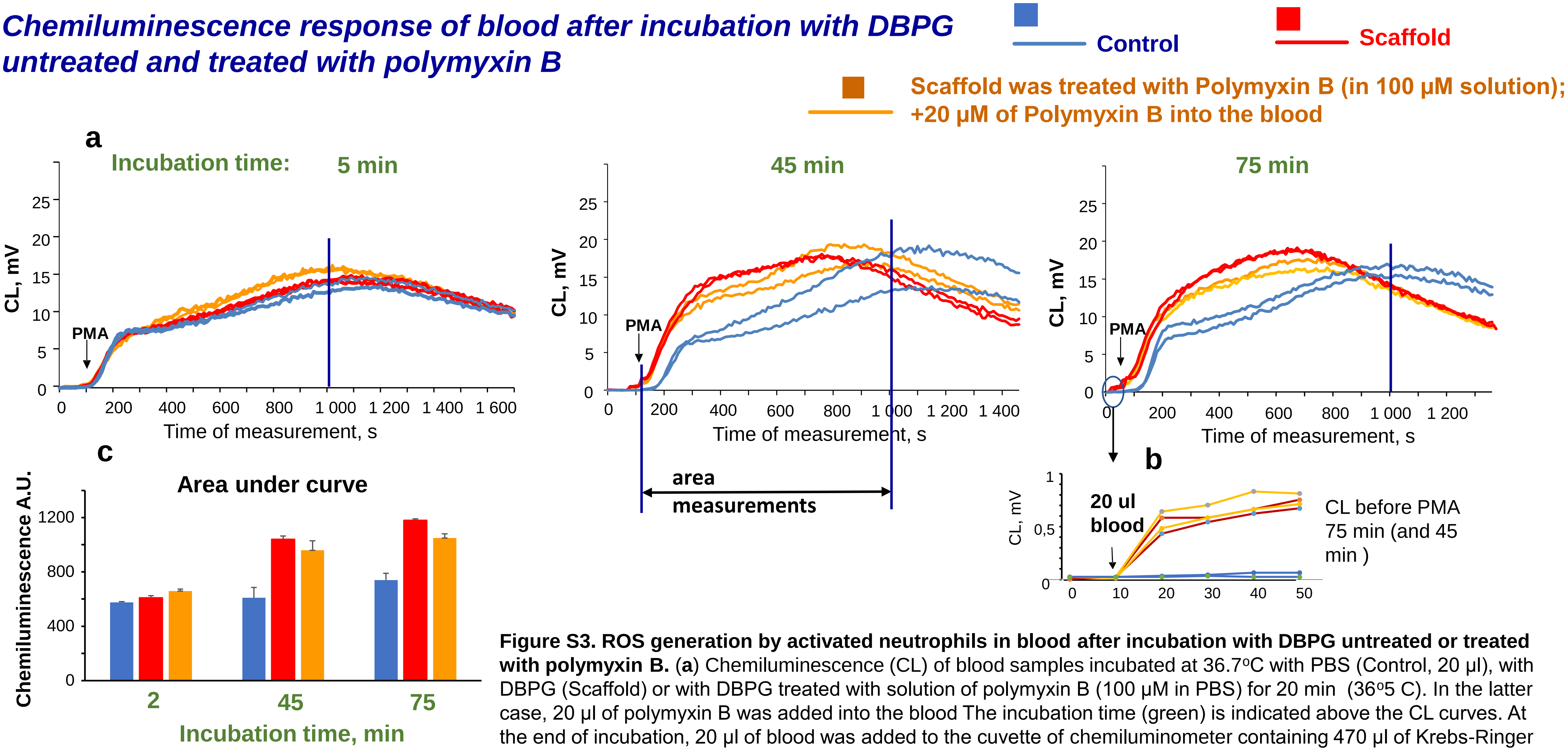

Figure S3. ROS generation by activated neutrophils in blood after incubation with DBPG untreated or treated with polymyxin B. (**a**) Chemiluminescence (CL) of blood samples incubated at 36.7ºC with PBS (Control, 20 µl), with DBPG (Scaffold) or with DBPG treated with solution of polymyxin B (100 µM in PBS) for 20 min (36º5 C). In the latter case, 20 µl of polymyxin B was added into the blood The incubation time (green) is indicated above the CL curves. At the end of incubation, 20 µl of blood was added to the cuvette of chemiluminometer containing 470 µl of Krebs-Ringer medium (with $NaHCO_3$ and $CaCl_2$) and 200 µM luminol.

The arrows indicate the time of addition of 100 nM PMA. Measurements for a given incubation time were carried out at 37ºC until the maximum was passed by all CL curves. The black vertical line indicates the cutoff time of measurements (1000 s) to calculate the area under CL curves. (**b**) CL of blood after 75 min incubation before addition of PMA. (**c**) Areas under the CL curves measured at different incubation time (indicated below the bars, green color).

Ascorbate and methionine attenuate luminol oxidation by activated neutrophils in blood

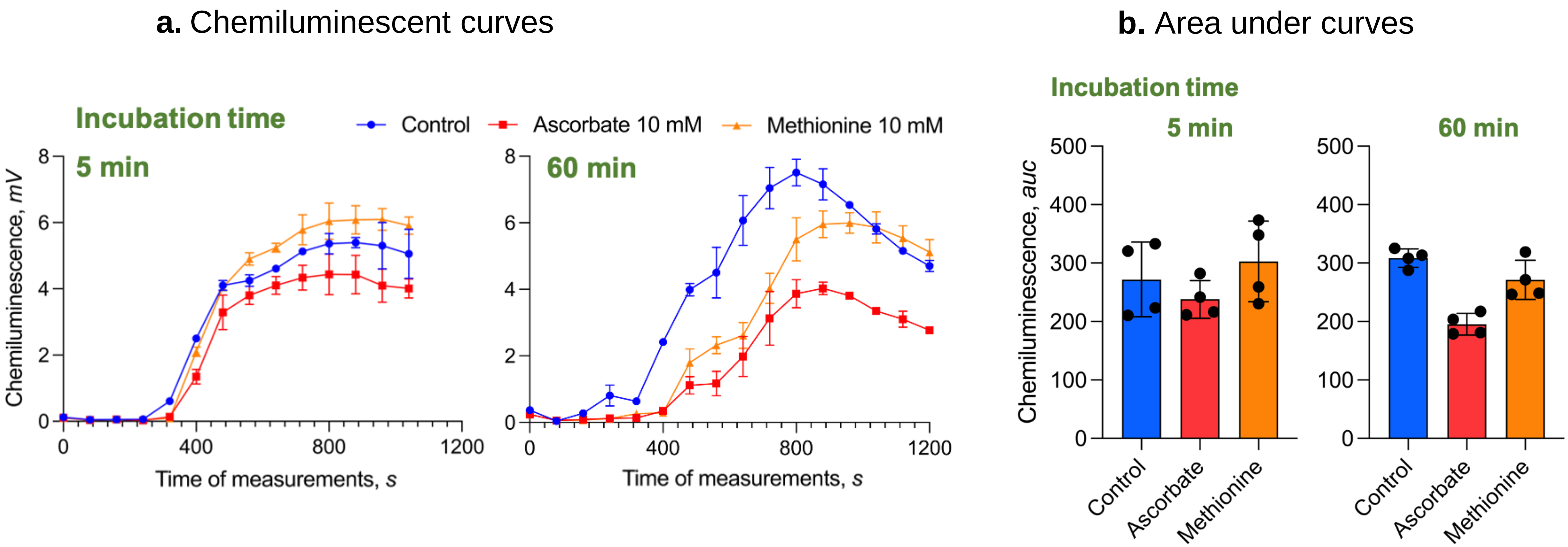

Figure S4. ROS generation by PMA-activated neutrophils in blood containing ascorbate (10mM) or methionine (10 mM).
(**a**) Chemiluminescence (CL) of blood samples incubated at 36.7°C with PBS (Control), with 10 mM ascorbate (Ascorbate) or with 10 mM methionine (Methionine) for 5 and 60 min (36°5 C). The incubation time (green) is indicated above the CL curves. At the end of incubation, 20 µl of blood was added to the cuvette of chemiluminometer containing 470 µl of Krebs-Ringer medium (with $NaHCO_3$ and $CaCl_2$) and 200 µM luminol. 100 nM PMA was added to activate neutrophils. Curves are means ± S.D of two replicates.

(**b**) Area under chemiluminescence curves were counted for 5 and 60 minutes of incubation. The incubation time (green) is indicated above bars. Data are means ± S.D., n=2 independent experiments.

Chemiluminescent response of blood after incubation with clinically used biomaterial: absorbable wound coverage ‘KOLLAGEN resorb’

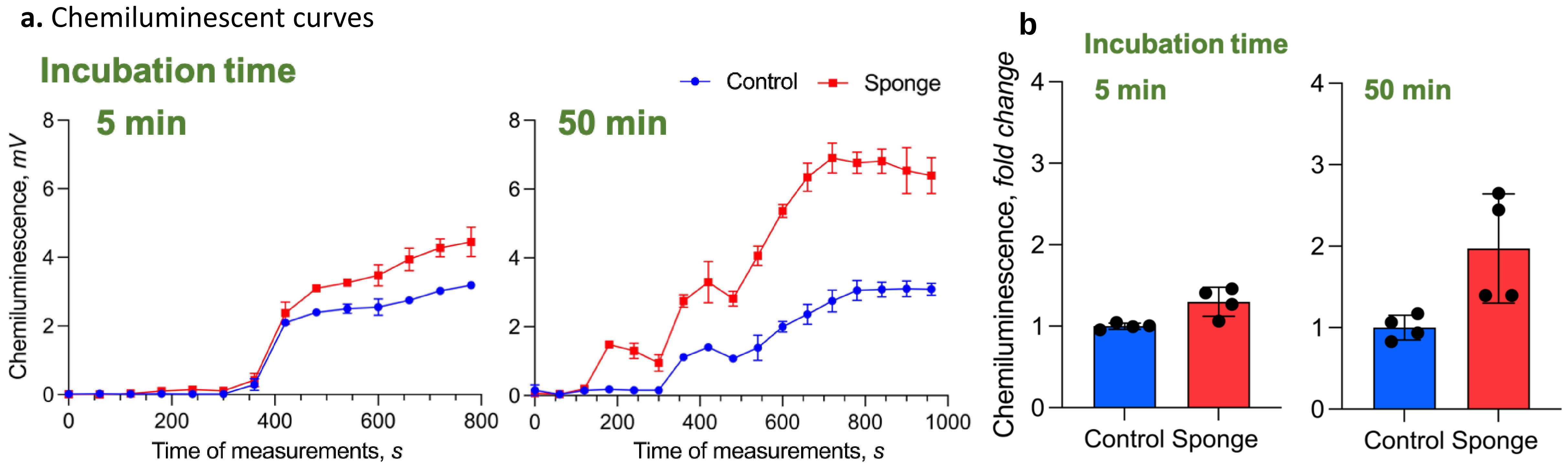

Figure S5. ROS generation by PMA-activated neutrophils in blood after blood incubation with collagen sponge.
(**a**) Chemiluminescence (CL) of blood samples incubated at 36.7ºC with PBS (Control) or with sponge scaffold for 5 and 60 min (36º5 C). The incubation time (green) is indicated above the CL curves. At the end of incubation, 20 µl of blood was added to the cuvette of chemiluminometer containing 470 µl of Krebs-Ringer medium (with $NaHCO_3$ and $CaCl_2$) and 200 µM luminol. 100 nM PMA was added to activate neutrophils. Curves are means ± S.D. of three replicates.

(**b**) Fold change of area under chemiluminescence curves were counted for 5 and 60 minutes of incubation. The incubation time (green) is indicated above bars. Data are means ± S.D., n=2 independent samples.

Confocal microscopy images of blood with low content of erythrocytes after incubation with DBPG

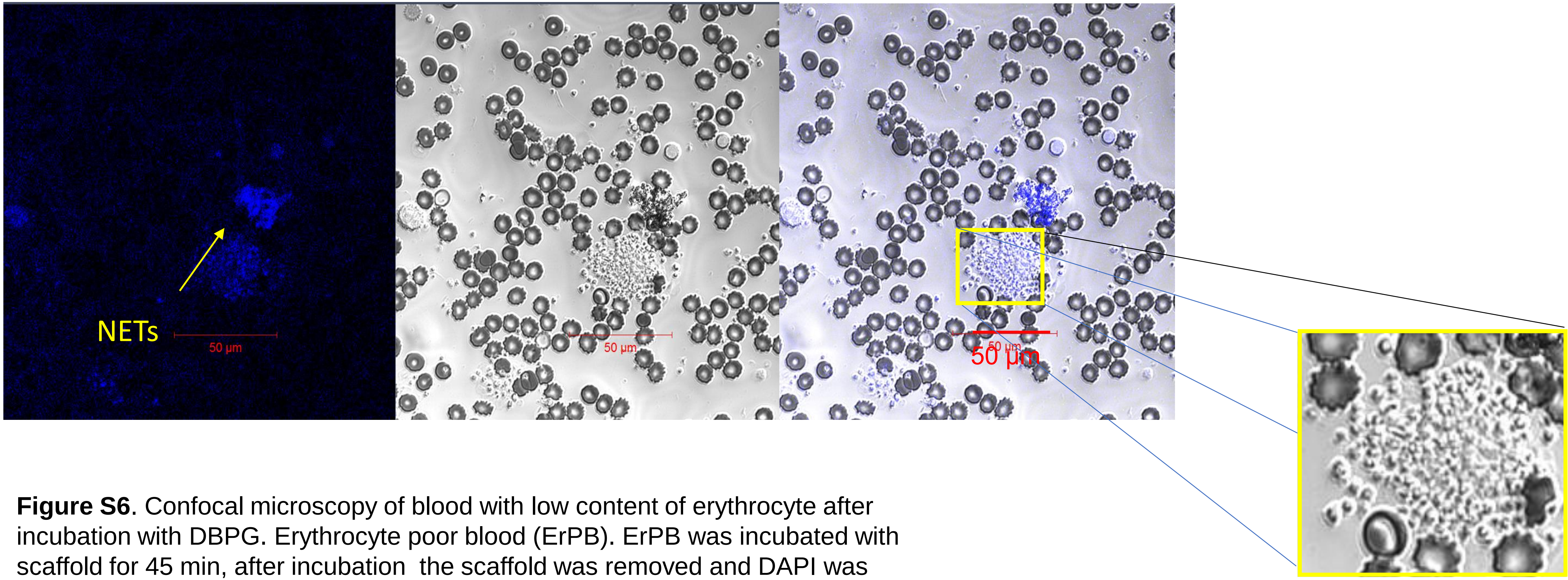

Figure S6. Confocal microscopy of blood with low content of erythrocyte after incubation with DBPG. Erythrocyte poor blood (ErPB). ErPB was incubated with scaffold for 45 min, after incubation the scaffold was removed and DAPI was added at concentration of 500 ng/ml. After 15 min incubation, cells were placed into confocal dish and images were acquired with ZEISS LSM 880 after cell adhesion to dish bottom. Most cells are erythrocytes. Some erythrocytes turned to echinocytes. Blue structures which are more then 20 µm looks like NETs.

Chemiluminescence of neutrophil suspension in plasma

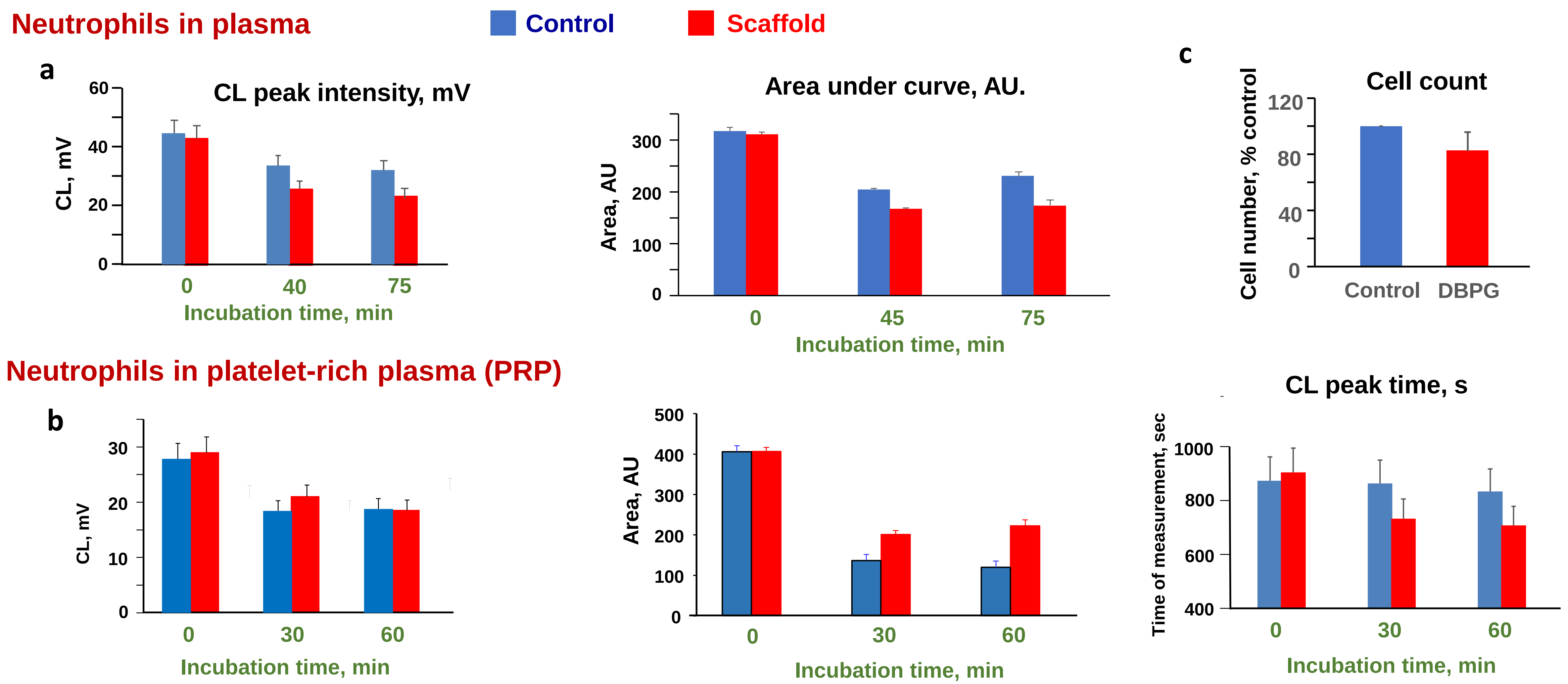

Figure S7. ROS generation by activated neutrophils in plasma (70% by volume) incubated with DBPG. Parameters of CL of neutrophil suspension in plasma (**a**) or in platelet rich plasma (**b**) incubated at 36.7^{o}C with PBS (Control, 20 μl) or with DBPG (Scaffold) for different time intervals. At the end of incubation, blood was added to the cuvette of chemiluminometer containing 485 μl of Krebs-Ringer medium (with $NaHCO_3$ and $CaCl_2$) and 200 μM luminol, in 2 min 100 nM PMA was added.
(**c**) Number of cells (% from control) in neutrophil suspension (0,25 mln cells per ml) in plasma (70%) incubated with DBPG for 40 min at 36.7^{o}C. DBPG (mg) : blood (μl) = 1:30.

Scheme of the interaction of scaffolds and neutrophils

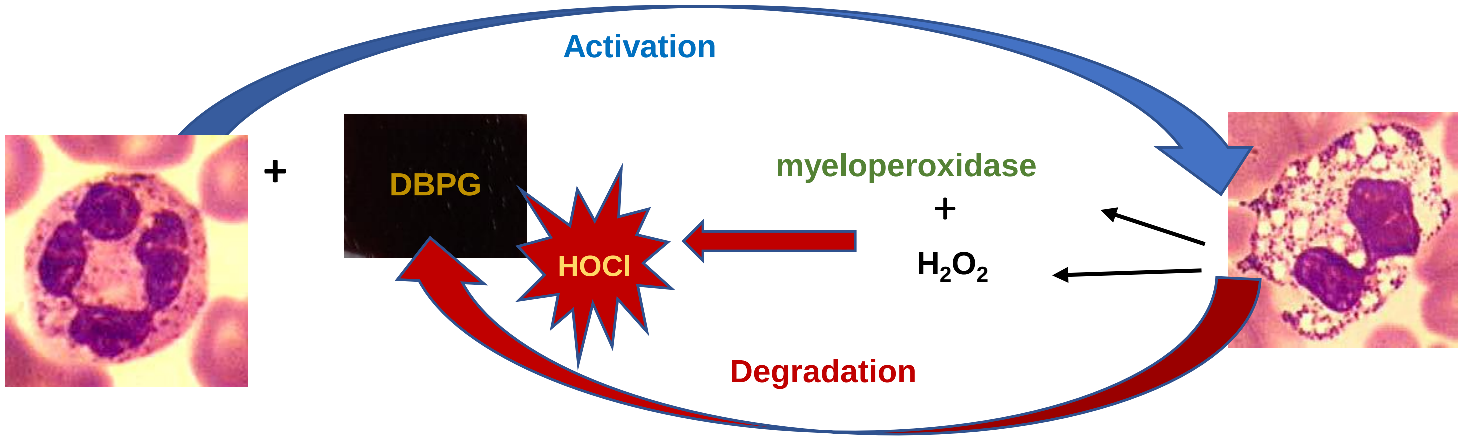

DBPG samples for FLIM measurements

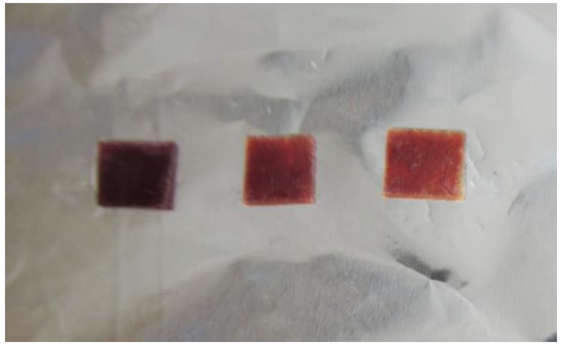

μmol NaOCl/mg DBPG	0	0.75	1.5
NaOCl mM	0.1	2-3	4-5

Figure S8. Scheme of the interaction of scaffolds and phagocytes:
Activation. Scaffold implantation causes activation of neutrophils.
Degradation. Free radicals and oxidants produced by activated phagocytes oxidize the surface of materials changing their structure and properties.
Oxidatively degraded scaffolds will affect the state of macrophages interacting with their surface and as a result change the polarization efficiency of macrophages in the M2 state.

Figure S9. Samples of DBPG after NaOCl treatment. Bleaching of DBPG accompanies the oxidation of scaffolds by hypochlorous acid.

Micro-aliqoutes of 0.5-1 μl NaOCl (1.7 M) were added to the scaffolds placed into 4 ml of buffer (PBS+50mM NaH_2PO_4, pH 7.3-7.4). NaOCl was added once or twice a day for 5-10 days up to the final concentration indicated in the Figure. Total incubation time was 12 days. Control sample was prepared three days before measurement and kept at 4ºC. FLIM images of the samples were acquired [44] .
